# Predicting nosocomial pneumonia of patients with acute brain injury in intensive care unit using machine-learning models

**DOI:** 10.3389/fmed.2025.1501025

**Published:** 2025-04-11

**Authors:** Junchen Pan, Zhen Yue, Jing Ji, Yongping You, Liqing Bi, Yun Liu, Xinglin Xiong, Genying Gu, Ming Chen, Shen Zhang

**Affiliations:** ^1^Department of Neurosurgery, The Affiliated BenQ Hospital of Nanjing Medical University, Nanjing, Jiangsu, China; ^2^Department of Neurosurgery, The First Affiliated Hospital of Nanjing Medical University, Nanjing, Jiangsu, China; ^3^Department of Information, The First Affiliated Hospital of Nanjing Medical University, Nanjing, Jiangsu, China; ^4^Department of Nursing, The Affiliated BenQ Hospital of Nanjing Medical University, Nanjing, Jiangsu, China; ^5^Department of Rehabilitation, The Affiliated BenQ Hospital of Nanjing Medical University, Nanjing, Jiangsu, China; ^6^Engineering Research Center of Health Service System Based on Ubiquitous Wireless Networks, Ministry of Education, Nanjing, China

**Keywords:** intensive care unit, area under the curve, acute brain injury, nosocomial pneumonia, machine-learning models

## Abstract

**Introduction:**

The aim of this study is to construct and validate new machine learning models to predict pneumonia events in intensive care unit (ICU) patients with acute brain injury.

**Methods:**

Acute brain injury patients in ICU of hospitals from January 1, 2020, to December 31, 2021 were retrospective reviewed. Patients were divided into training, and validation sets. The primary outcome was nosocomial pneumonia infection during ICU stay. Machine learning models including XGBoost, DecisionTree, Random Forest, Light GBM, Adaptive Boost, BP, and TabNet were used for model derivation. The predictive value of each model was evaluated using accuracy, precision, recall, F1-score, and area under the curve (AUC), and internal and external validation was performed.

**Results:**

A total of 280 ICU patients with acute brain injury were included. Five independent variables for nosocomial pneumonia infection were identified and selected for machine learning model derivations and validations, including tracheotomy time, antibiotic use days, blood glucose, ventilator-assisted ventilation time, and C-reactive protein. The training set revealed the superior and robust performance of the XGBoost with the highest AUC value (0.956), while the Random Forest and Adaptive Boost had the highest AUC value (0.883) in validation set.

**Conclusion:**

Machine learning models can effectively predict the risk of nosocomial pneumonia infection in patients with acute brain injury in the ICU. Despite differences in populations and algorithms, the models we constructed demonstrated reliable predictive performance.

## Introduction

Acute brain injury (ABI) encompasses a range of neurological disorders that can result in acute functional deficits, including ischemic or hemorrhagic stroke, subarachnoid hemorrhage from aneurysms, and traumatic brain injury, causing approximately 12 million deaths annually (1, 2). Due to the uncertain long-term functional prognosis of ABI, patients often have prolonged hospital stays, and the majority require endotracheal intubation for airway protection, along with mechanical ventilation and intracranial pressure reduction. Additionally, ABI is associated with immune system alterations mediated through inflammation and the autonomic nervous system, which may increase susceptibility to infections during and after hospitalization (3–5). In ABI patients who suffer from nosocomial infections, a common source of infection is the respiratory system, including ventilator-associated pneumonia and hospital-acquired pneumonia. Studies have shown that nosocomial pneumonia significantly prolongs patients’ hospital stay and increases mortality and disability rates (3, 6).

Studies have indicated a significant association between the severity of ABI, chest trauma, smoking history, substance abuse, as well as interventions such as transfusion, sedation, and the need for tracheostomy, with the risk of nosocomial pneumonia (7–11). Given that nosocomial pneumonia can be considered a continuum of a single disease, describing its epidemiology and influencing factors is clinically valuable (12, 13). This can aid in better formulating preventive and management measures for nosocomial pneumonia in clinical practice. Existing pneumonia prediction scoring tools in clinical practice, such as CPIS score, A2DS2 score, and AIS-APS score, have certain limitations, primarily due to underutilization of classification information, leading to information loss (14–16).

Recently, artificial intelligence (AI) has rapidly developed in the field of medicine, with machine learning being the most widely used AI method. Machine learning models generate personalized probabilities of events for patients. Additionally, ML models can capture complex non-linear relationships in medical data, making full use of clinical information (17). However, there is currently no effective tool for rapidly predicting the occurrence of nosocomial pneumonia in ICU patients with ABI. This study aims to collect factors related to the occurrence of nosocomial pneumonia in neurosurgical ICU patients with ABI through retrospective analysis, and ultimately construct a predictive model for nosocomial pneumonia in ICU patients with ABI.

## Materials and methods

### Study setting

The patients admitted to the Neurosurgery Departments of Jiangsu Provincial People’s Hospital and Benq Hospital from January 1, 2020, to December 31, 2021 were selected as the training set. To validate the model’s generalization ability, patients from three healthcare systems, including Suqian First People’s Hospital, Nanjing Jiangning Hospital, and Jiangsu Provincial People’s Hospital, were selected as the external validation set. This study obtained approval from the Institutional Review Board of the research center. Given the retrospective design of this study, the requirement for obtaining informed consent from patients was waived. This study was reported in accordance with the Transparent Reporting of a Multivariable Prediction Model for Individual Prognosis or Diagnosis (TRIPOD) reporting guideline (18).

### Inclusion and exclusion criteria

Patients were included if they met the following criteria: (1) aged between 18 and 80 years old; (2) admitted to the Neurosurgery ICU with ABI, including cerebral hemorrhage, trauma, vascular disease, or tumor; (3) hospital stay longer than 48 h; (4) received standard treatment during hospitalization, including surgical and conservative treatment. Patients with the following characteristics were excluded: (1) occurrence of pulmonary infection within 48 h of ICU admission or hospitalization; (2) patients who did not receive standardized postoperative treatment; (3) patients with immunodeficiency diseases; (4) patients with severe chronic heart, lung, kidney, or other organ diseases. Severe chronic heart disease included end-stage heart failure, severe cardiomyopathies, and complex congenital heart diseases that demand continuous medical support and substantially affect the patient’s physiological function and prognosis; (5) patients with concomitant malignant tumors in other organs; (6) patients for whom data were unavailable.

### Data collection

Collect demographic characteristics, vital signs, ventilator parameters, sputum culture results, blood test results, imaging findings, treatment, and prognosis data from the hospital electronic medical record system for ICU patients with acute brain injury, encompassing 61 dimensions. Nosocomial infections are defined as those that occur more than 48 h after hospital admission, and the diagnosed pneumonia based on clinical presentation, blood-related examinations, vital signs, radiological examinations, and sputum culture.

### Statistical analysis

We used SPSS 26.0 and Python 3.7 for data analysis. Data cleaning was performed, including the removal of variables not included in the statistical analysis, missing values (with a missing rate exceeding 80%), and continuous values containing character groups. Subsequently, duplicate data entries were removed, and the cleaned data were exported. For variables with a missing rate of less than 80%, we used the Multivariate Imputation by Chained Equations (MICE) algorithm for imputation. MICE is a multiple imputation method that iteratively models each variable with missing values as a function of the other variables in the dataset. This approach allows us to capture the complex relationships between variables, which is essential in our medical dataset. By using MICE, we aimed to generate more accurate imputations and preserve the data’s underlying structure. After imputation, the dataset was further processed for subsequent analysis. The training set were randomly divided into training subset and internal validation subset at a ratio of 7:3. Out of the total 280 patients, 196 patients were assigned to the training set, and 84 patients were included in the validation set. Continuous variables that followed a normal distribution were presented as mean ± standard deviation, while those not following a normal distribution were presented as median and interquartile range. Categorical variables were presented as counts and proportions. Independent sample t-tests were used to compare normally distributed continuous variables, Mann–Whitney *U* tests were used for non-normally distributed continuous variables, and chi-square tests were used for comparisons between categorical variables. A *p*-value <0.05 was considered statistically significant.

Subsequently, the selected variables were used to build seven machine learning models, including the XGBoost model, DecisionTree model, Random Forest model, Light GBM model, Adaptive Boost model, BP model, and TabNet model. Selecting these models owing to their unique advantages in handling complex non-linear relationships within medical data. Ensemble models are well-equipped to capture interactions among multiple variables, which is crucial when predicting nosocomial pneumonia in ICU patients with ABI. Receiver operating characteristic (ROC) curves were plotted, and the area under the curve (AUC) was calculated. Furthermore, the performance of each model was evaluated by comparing their accuracy, precision, recall, and F1-score on both the internal validation subset and external validation set.

SPSS 26.0 was employed for initial data exploration and basic statistical tests, while Python 3.7 was used for data cleaning, machine-learning model construction, and performance evaluation. The *pandas* library in Python was crucial for data manipulation tasks. For building the machine-learning models, we relied on several libraries. The *scikit-learn* library was extensively used. The XGBoost model was implemented using the *xgboost* library, which offers efficient algorithms for gradient-boosting. The DecisionTree model was built using the *DecisionTreeClassifier* class from *scikit-learn*, and the Random Forest model was created using the *RandomForestClassifier* class in the same library. The Adaptive Boost model was implemented using the *AdaBoostClassifier* class from *scikit-learn*. The Light GBM model was built with the *lightgbm* library. The BP model was implemented using the *Keras* library, which is a high-level neural network API running on top of *TensorFlow*. The TabNet model was created using the *pytorch-tabnet* library designed for tabular data. For performance evaluation, functions from *scikit-learn* were used to assess performance evaluation.

## Results

This study included a total of 280 ABI patients, and the baseline characteristics of the patients are shown in Table 1. A total of 67 patients with nosocomial pneumonia, while the remaining 213 patients without nosocomial pneumonia. There were no significant differences between groups for age (*p* = 0.899), sex (*p* = 0.176), history of heart disease (*p* = 0.208), and patients received chemotherapy or immunosuppressive therapies (*p* = 0.074). However, we noted significant differences between groups for smoking history (*p* = 0.046), diabetes (*p* < 0.001), and stroke history (*p* = 0.012).

**Table 1:** The baseline characteristics of included patients.

Variables	Nosocomial pneumonia		*P-*value
	Yes (*n* = 67)	No (*n* = 213)	
Age	60.15	52.16	0.899
Sex (%)			0.176
Male	44	158	
Female	23	55	
Smoking history			0.046
Yes	16	79	
No	51	134	
Diabetes			<0.001
Yes	14	12	
No	53	201	
Heart disease			0.208
Yes	5	8	
No	62	205	
Stroke			0.012
Yes	8	8	
No	59	205	
Chemotherapy or immunosuppressive therapies			0.074
Yes	1	0	
No	66	213	

**Figure 1:**
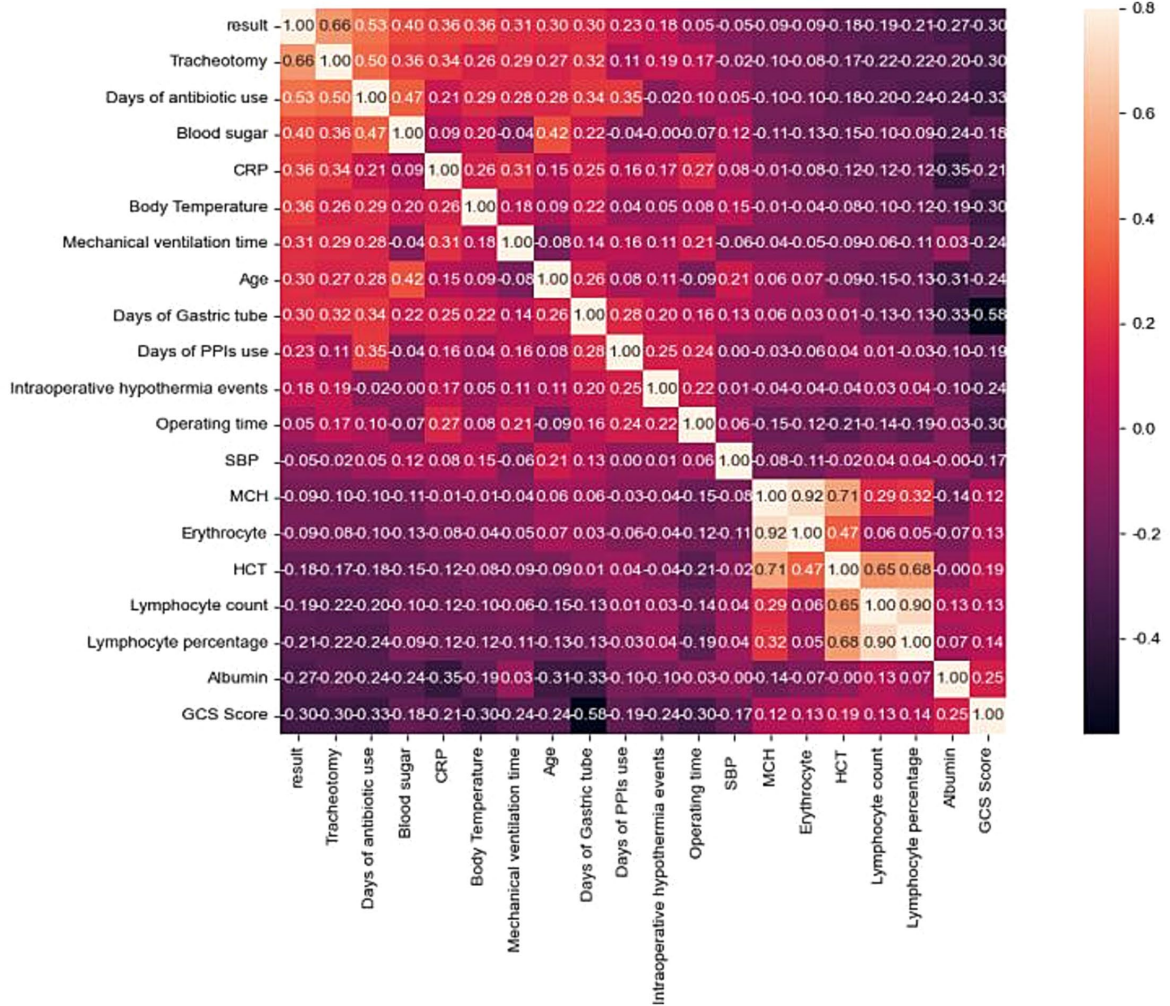
Pearson correlation heatmap between variable.

**Figure 2:**
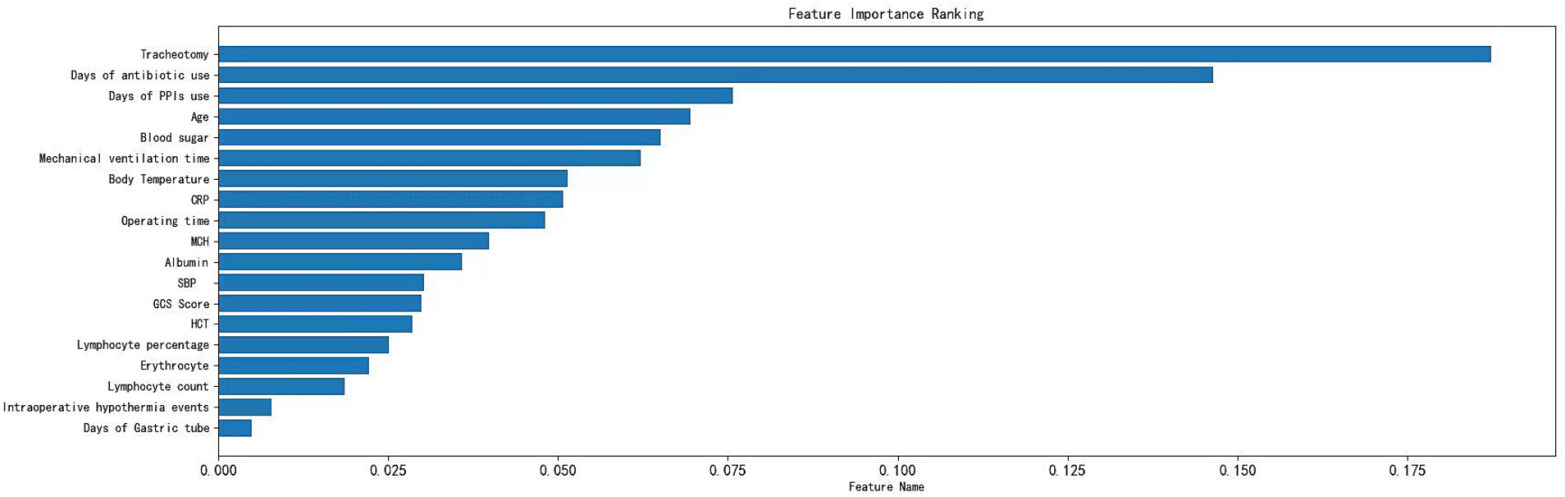
Variable importance of features included in the machine learning algorithm for prediction of nosocomial pneumonia in ABI patients.

A total of 2,953 data points were collected from 280 patients, including 24 qualitative features and 35 quantitative features (Appendix). Fifty-nine variables were analyzed through correlation heatmap analysis and random forest-based feature selection. The most significant predictors were subsequently entered into multiple logistic regression modeling. Pearson correlation heatmap analysis was used for correlation analysis, revealing that the duration of tracheostomy was the most significant associated factor for pneumonia occurrence in ABI patients (Figure 1). In addition, the results of the Light Gradient Boosting Machine algorithm showed that the top 20 important indicators were tracheostomy duration, duration of antibiotic use, blood sugar, age, duration of mechanical ventilation, CRP, GCS score, body temperature, lymphocyte percentage, lymphocyte count, gastric tube, albumin, PPi, intraoperative hypothermia, total protein, erythrocyte count, average hemoglobin concentration, hematocrit, operation time, and mean corpuscular volume (Figure 2).

**Figure 3:**
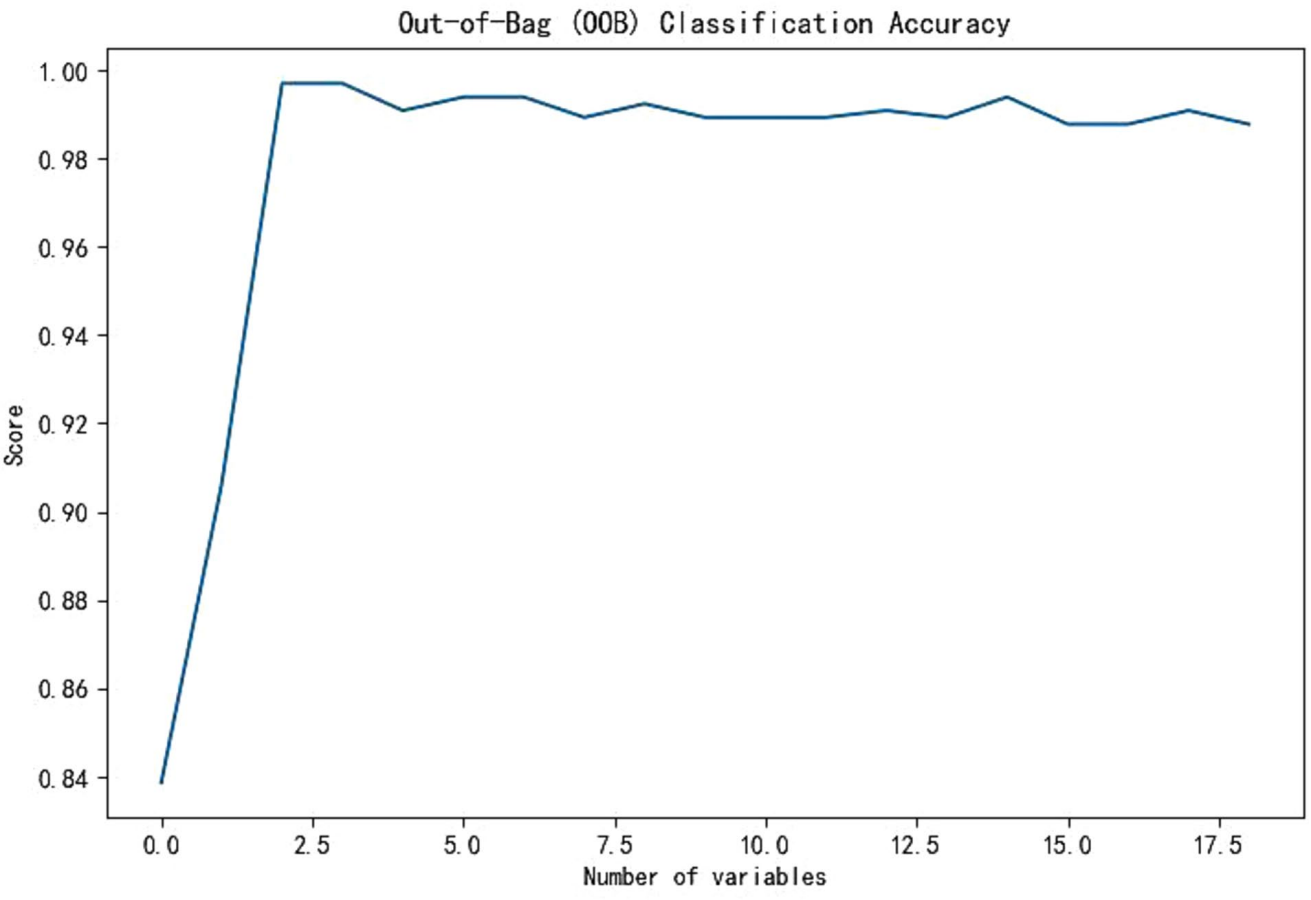
Accuracy chart of out-of-pocket data.

**Figure 4:**
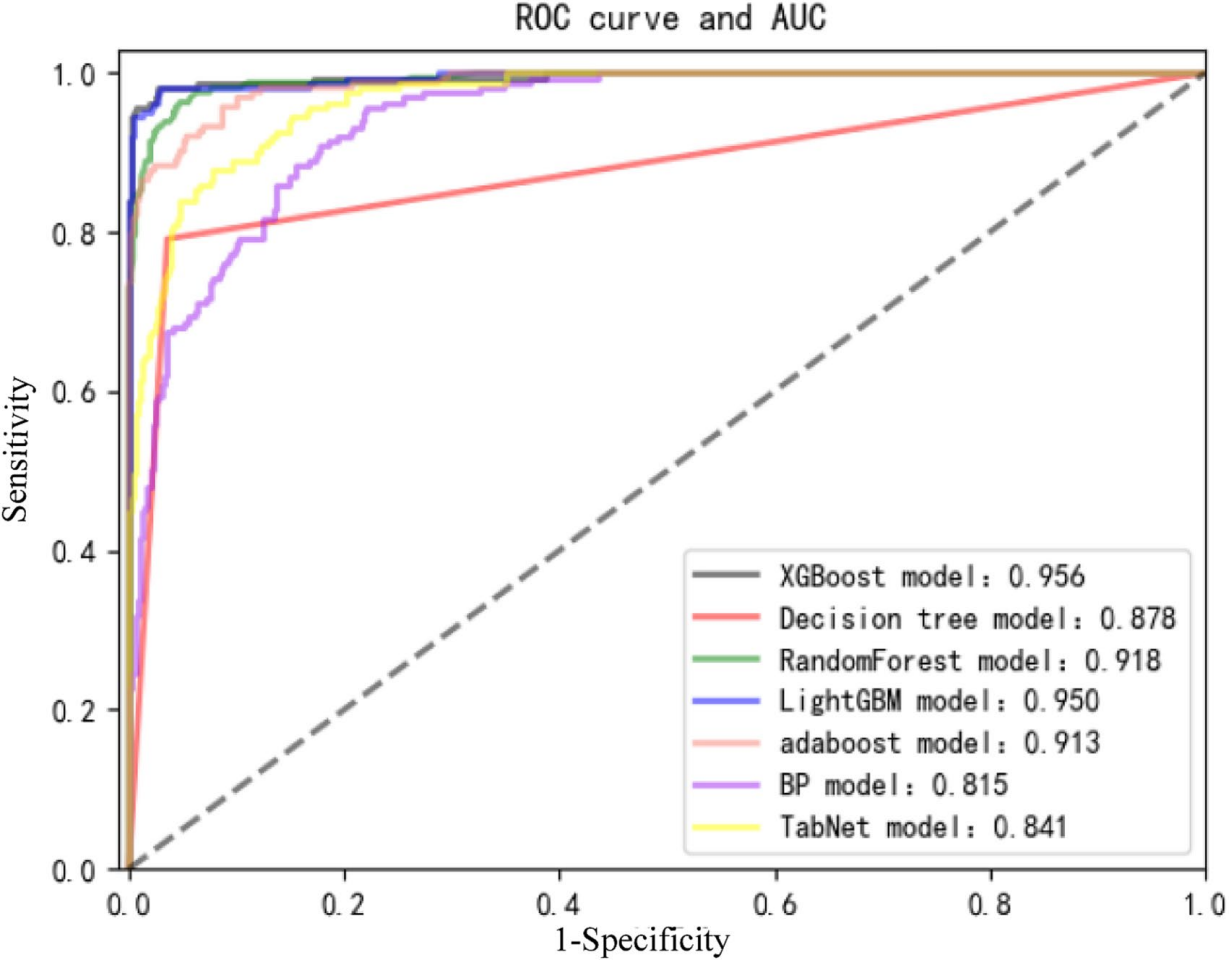
Receiver operating characteristic (ROC) curve and AUC among the 7 algorithm models.

**Figure 5:**
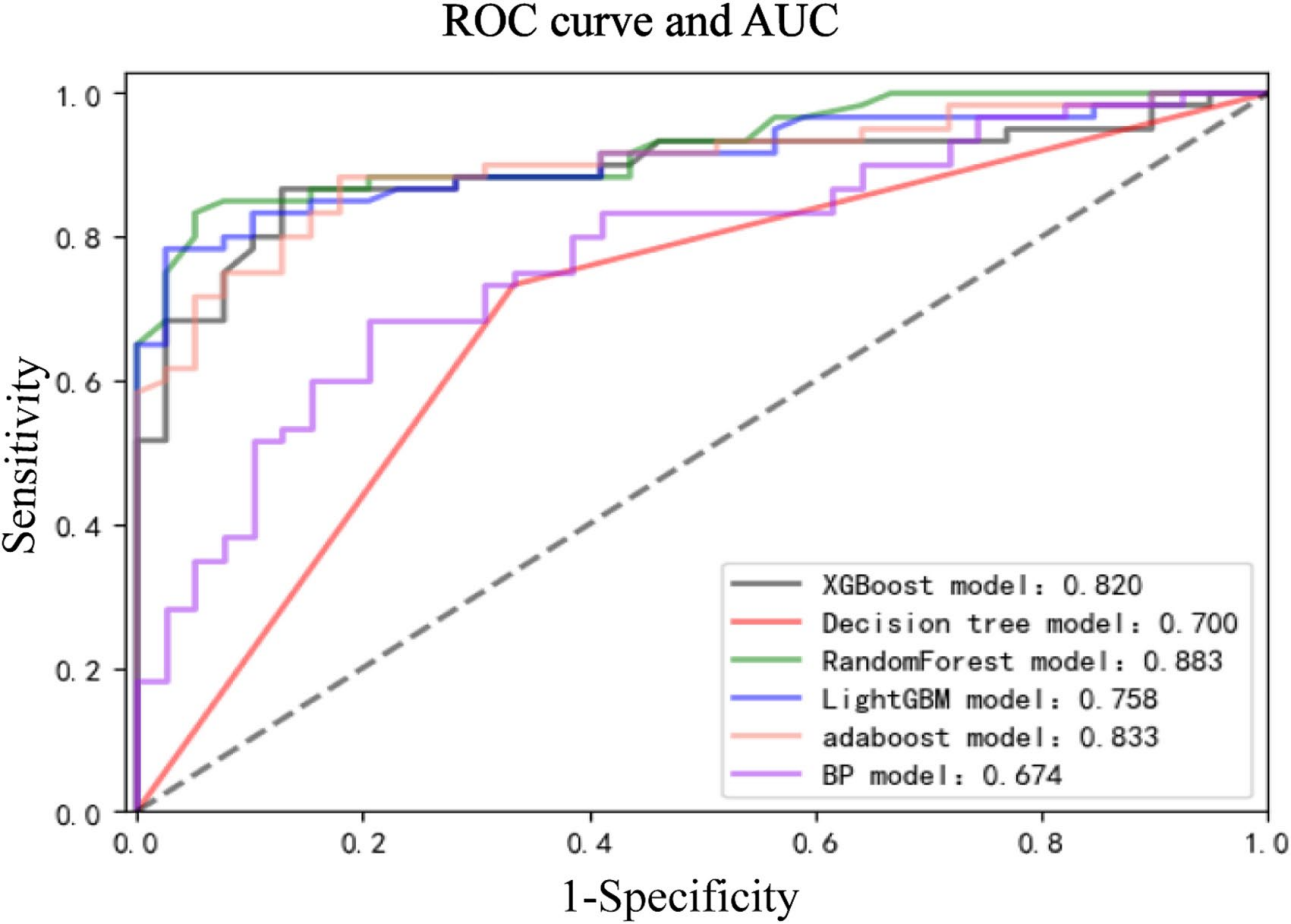
Receiver operating characteristic curve and AUC among the external validation set.

Through the cross-validation accuracy curve, it can be observed that when the number of features is 5. It should be noted that while the out-of-Bag (OOB) error score was relatively high at this point, our objective was to find the optimal number of features for overall model performance. The OOB error is just one of the many factors to consider. By examining the OOB error in combination with other performance metrics such as accuracy, precision, recall, F1-score, and AUC, we aimed to select a feature set that would provide the best generalization and predictive ability for the model. A slightly higher OOB error might be tolerated if the model shows superior performance in other aspects, which is crucial for practical application in predicting nosocomial pneumonia in ICU patients with ABI. Employing a stepwise method, with 5 features including tracheostomy time, duration of antibiotic use, blood sugar level, duration of mechanical ventilation, and CRP were applied to establish prediction model (Figure 3). Among the seven machine-learning algorithms evaluated, the XGBoost and Light GBM models demonstrated relatively high AUC values, indicating their strong discriminatory power in predicting nosocomial pneumonia in ICU patients with ABI (Table 2 and Figure 4). The external validation set found XGBoost showed highest precision (0.96), while Random Forest and Adaptive Boost models showed highest Light GBM (AUC: 0.883) (Table 2 and Figure 5). These high-performing models based on AUC values can potentially play a crucial role in clinical decision-making. A model with a high AUC can assist clinicians in early identification of patients at high risk of nosocomial pneumonia, enabling timely intervention and potentially improving patient outcomes.

**Table 2:** The predictive performance among the constructed models.

Algorithmic	Training set				Validation set			
	Precision	Recall rate	F1-score	AUC	Precision	Recall rate	F1-score	AUC
XGBoost	0.98	0.96	0.97	0.956	0.96	0.96	0.96	0.820
Decision tree	0.91	0.88	0.89	0.878	0.92	0.92	0.92	0.700
Random Forest	0.96	0.92	0.94	0.918	0.94	0.94	0.94	0.883
Light GBM	0.98	0.95	0.96	0.950	0.95	0.95	0.96	0.758
Adaptive Boost	0.96	0.91	0.93	0.913	0.93	0.93	0.93	0.883
BP network	0.86	0.81	0.83	0.815	0.84	0.84	0.85	0.758
TabNet	0.90	0.84	0.86	0.841	0.88	0.88	0.89	0.674

### Model visualization

**Figure 6:**
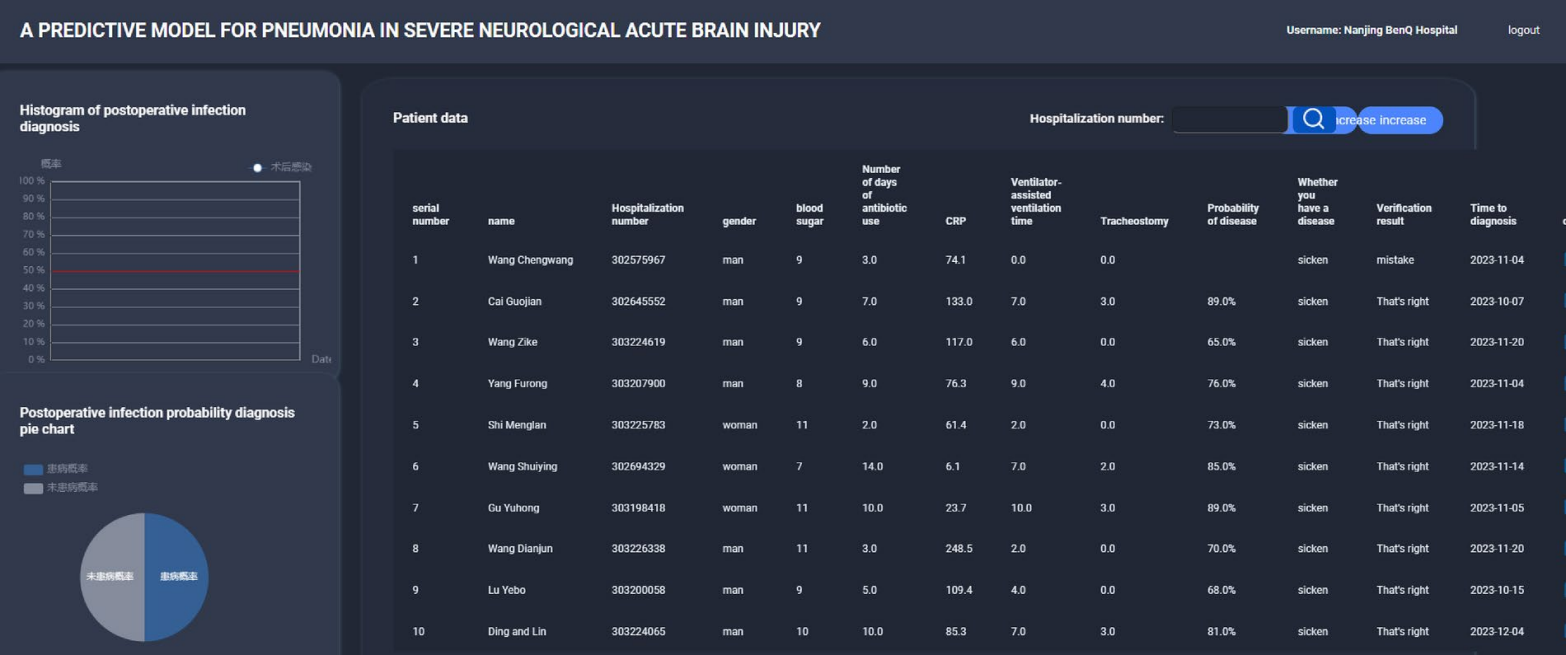
The diagnosis model of XGBoost is used to predict the infection risk.

Leveraging an XGBoost-based diagnostic model, our data platform conducts daily predictions of infection risk (Figure 6). Although we cannot provide a direct link to the platform due to privacy and security reasons, the platform functions in a manner similar to the interactive capabilities of the ‘*shiny*’ package in R software. Users input the five key parameters: tracheostomy time, duration of antibiotic use, blood glucose levels, the length of time on ventilator support, and the CRP value. The platform then processes these inputs using the underlying XGBoost-based algorithm. The output is presented in a dynamic way. A pie chart shows the real – time distribution of incidence probabilities, giving users an immediate understanding of the patient’s risk status. Additionally, a trend chart is available for each patient, which can be used to track the progression of the predicted risk over time. This visualization aims to assist clinicians in making more informed decisions regarding patient care.

## Discussion

Currently, clinicians typically rely on clinical, radiological, and laboratory indicators to diagnose pneumonia and initiate empirical antibiotic therapy (19). However, early pneumonia is insensitive, radiological radiation causes some harm to patients, and clinical diagnosis of pathogenic microorganisms has some lag. Existing scoring systems do not cover all individual factors of patients and have low accuracy. Considering the impracticality of existing prediction models and the flexibility of machine learning methods in selecting predictive variables and transformation algorithms. This study employed machine learning to construct a predictive model for nosocomial pneumonia infection in ABI patients in the ICU. The study included 280 patients from multiple hospitals for model construction and validation. The results revealed that among the machine learning models constructed based on tracheostomy time, duration of antibiotic use, blood sugar level, duration of mechanical ventilation, and CRP, the XGBoost model exhibited the best overall performance. It achieved a precision of 98% and an AUC of 95.6% in predicting postoperative pneumonia in ABI patients. Similarly, in the external validation set, we observed the highest precision with the XGBoost model.

Several studies have already constructed a prediction model for pneumonia in patients with brain injury using a machine learning approach (20, 21). Zheng et al. (20) identified 468 patients with spontaneous intracerebral hemorrhage (sICH) and identified six independent variables, including nasogastric feeding, airway support, unconscious onset, surgery for external ventricular drainage, larger sICH volume, and ICU stay, and the prediction model constructed based on these variables could effectively predict stroke-associated pneumonia in patients with sICH. Lee et al. (21) identified 5,754 hospitalized stroke patients, and found random survival forest model showed superior discriminative ability for predicting post-stroke pneumonia. However, there are currently no studies that have constructed predictive models for nosocomial pneumonia infection in ABI populations in the ICU. Therefore, we adopted a machine learning approach to construct a predictive model for nosocomial pneumonia infection in ABI populations in the ICU, which can manage missing information without the need for imputation or preprocessing and has strong clinical applicability.

To further evaluate the performance of our proposed machine-learning models, we compared them with existing clinical tools, namely CPIS, A2DS2, and AIS-APS scores. With a cut-off value of CPIS ≥ 3, in critically ill patients, the AUC of CPIS for predicting ventilator-associated pneumonia was found to be 59% (22). The AUC of the A2DS2 model for predicting stroke-associated pneumonia was 85% (23). At the same time, the AUC of the AIS-APS score for predicting ischemic stroke-associated pneumonia was 87% (24). The optimal model constructed in this study had a higher AUC than the previous prediction models in both the training set and the validation set, highlighting the potential advantages of our machine-learning-based approach in predicting nosocomial pneumonia in ICU patients with ABI.

The constructed prediction model based on tracheostomy time, duration of antibiotic use, blood sugar level, duration of mechanical ventilation, and CRP showed high predictive performance. The potential reasons for this could explained by: (1) long-term endotracheal intubation and mechanical ventilation may lead to respiratory mucosal injury and inflammatory responses, thereby affecting local immune function, weakening the clearance ability against pathogenic microorganisms, and increasing the likelihood of infection occurrence (25); (2) long-term use of antibiotics may lead to bacterial resistance to drugs and cause a series of adverse reactions, such as disruption of intestinal flora balance, liver and kidney damage, thereby increasing the risk of infection (26); (3) high blood sugar may lead to immune suppression and exacerbate inflammation, which can reduce the body’s ability to clear pathogens, trigger the release of inflammatory mediators, increase inflammation in lung tissues, and thus provide an optimal environment for bacterial infection (27); (4) various factors related to mechanical ventilation, such as positive end-expiratory pressure (PEEP), tidal volume, and FiO2 levels, can contribute to lung injury and inflammation, further predisposing patients to ventilator-associated pneumonia (28); and (5) elevated levels of CRP often reflect the inflammatory status of the body. The exacerbation of pulmonary inflammation may lead to tissue damage in the lungs and provide a favorable environment for the growth of pathogenic microorganisms, thereby increasing the risk of nosocomial pneumonia (29).

The results of this study found that the XGBoost model had the best performance in predicting nosocomial pneumonia infection among ABI patients in the ICU. The XGBoost model is commonly used for data mining. It is less prone to overfitting on limited datasets and has lower processing requirements compared to deep learning methods, yet it performs well under various variable conditions (30). Compared to deep learning models, our study objectives and dataset do not require the extraction of larger datasets. This also explains why the XGBoost model is most suitable. Combining the results of this study, a data platform based on the XGBoost diagnostic model can be constructed. Patient basic information, along with parameters including tracheostomy time, duration of antibiotic use, blood sugar level, duration of mechanical ventilation, and CRP, are input into the platform card. Through the platform calling the algorithm interface, diagnostic results can be displayed on the platform, showing the probability of occurrence in real-time, making the diagnosis more intuitive and better guiding clinical treatment.

Several shortcoming of this study should be mentioned. Firstly, this study was retrospectively designed, and the research results may be affected by recall bias and confounding bias. Secondly, the definition of nosocomial pneumonia varies across different research centers, which may affect the predictive ability of the constructed prediction models. Thirdly, not all variables are balanced across all research centers, which may introduce bias into the results. Although consistent results were obtained based on these unbalanced variables, the impact of heterogeneity should not be underestimated.

## Conclusion

This study derived a predictive model for nosocomial pneumonia infection in ICU patients with ABI using machine learning techniques from multiple centers, and conducted multiple validations to obtain effective and robust confirmation. The results indicate that machine learning-based models can more accurately predict the risk of nosocomial pneumonia infection in ICU patients with ABI, aiding in the early identification and intervention of nosocomial pneumonia infection. It should be noted that our study has limitations related to the relatively small dataset size in the context of having over 50 variables. A small dataset may increase the risk of overfitting, as the machine-learning models may adapt too closely to the specific features of this limited sample, leading to poor generalization to new data. Moreover, it may not fully represent the entire spectrum of variability in the population of ICU patients with ABI, thus potentially limiting the generalizability of our findings. To address these limitations, future research could consider expanding the sample size. Multi-center studies could be conducted to gather a larger and more heterogeneous dataset, which would likely improve the stability and generalizability of the predictive models.

## Data Availability

The raw data supporting the conclusions of this article will be made available by the authors, without undue reservation.
