# Supplementary material for: Predicting nosocomial pneumonia of patients with acute brain injury in intensive care unit using machine-learning models
Source: Front Med (Lausanne). 2025 Apr 11;12:1501025. doi: 10.3389/fmed.2025.1501025 (PMC12021828; doi:10.3389/fmed.2025.1501025)
Supplement: Supplementary file 1 [file Table_1.docx]

**List of variables:** age, sex, smoking history, diabetes, heart disease, stroke, chemotherapy or immunosuppressive therapies, tracheotomy, days of antibiotic use,

blood sugar, CRP, body temperature, mechanical ventilation time, days of gastric tube, days of PPIs use, intraoperative hypothermia events, operating time, SBP, MCH, erythrocyte, HCT, lung diseases, GSC score, difficulty in swallowing, days of urethral catheter, intubation, central vein, surgery type, hypotensive events, vasoactive drugs, intraoperative blood transfusion, postoperative blood transfusion, days of hormone use, use of analgesic and sedative drugs, mannitol, pulmonary rehabilitation, pulmonary infection, white blood cells, hemoglobin, platelets, mean corpuscular volume, mean corpuscular hemoglobin concentration, red blood cell distribution width SD, red blood cell distribution width CV, neutrophil percentage, lymphocyte percentage, monocyte percentage, eosinophil percentage, basophil percentage, neutrophil count, lymphocyte count, monocyte count, eosinophil count, basophil count, total protein, albumin, globulin, heart rate, respiration
